# Component-Resolved and Multiplex-Specific IgE Diagnostics: Utility in Anaphylaxis and Beyond

**DOI:** 10.3390/children12070933

**Published:** 2025-07-16

**Authors:** Mirjana Turkalj, Ivana Banić, Gordana Fressl Juroš

**Affiliations:** 1 Department of Allergy and Clinical Immunology, Srebrnjak Children’s Hospital, HR-10000 Zagreb, Croatia; mturkalj@bolnica-srebrnjak.hr; 2 School of Medicine, Catholic University of Croatia, HR-10000 Zagreb, Croatia; 3 Faculty of Medicine, J.J. Strossmayer University of Osijek, HR-31000 Osijek, Croatia; 4 Department of Medical Research, Srebrnjak Children’s Hospital, HR-10000 Zagreb, Croatia; 5 Synevo Croatia, Medicover, HR-10000 Zagreb, Croatia; gordana.fressl-juros@synevo.com

**Keywords:** anaphylaxis, allergen-specific immunoglobulin E (IgE), component-resolved diagnostics, singleplex sIgE assay, multiplex IgE assays, molecular diagnostics in allergy

## Abstract

The diagnosis of allergic diseases and anaphylaxis is complex and encompasses a broad spectrum of in vitro and in vivo diagnostic tests. The choice of diagnostic tests is related to the presumed pathophysiological mechanism of the allergic reaction. In the past decade the implementation of component-resolved diagnostics (CRD) into clinical practice has significantly improved the depicting of sensitization profiles, which has aided in the assessment of clinically relevant allergen components that are associated with true allergy, as well as the levels of risk of severe anaphylactic reactions. Recently, multiplex-specific immunoglobulin E (IgE) platforms have emerged for better selection of patients at risk for anaphylaxis and have improved the selection criteria for patients undergoing allergen immunotherapy, including novel regimes such as oral immunotherapy. This review describes the advantages of the utilization of component-resolved diagnostics and multiplex assays in clinical settings, especially in cases of anaphylaxis when no clear trigger is recognized or where multiple culprits are suspected. As multiplex component-resolved diagnostics becomes more readily available globally and with the use of novel approaches, CRD will certainly be a crucial tool in personalized and individually tailored management plans and reduce the financial burden of anaphylaxis.

## 1. Introduction

Anaphylaxis is an acute, systemic, life-threatening hypersensitivity reaction caused by specific and non-specific triggers. Specific triggers include a number of different allergens such as foods, insect stings and medications, while non-immunologic triggers directly activate mast cells and basophils. The lifetime prevalence of anaphylaxis ranges from 0.05 to 5.1% worldwide, with a rising trend in the last 20 years. The lifetime prevalence of anaphylaxis in Europe is estimated at approximately 3%, with up to 30% of anaphylactic reactions requiring hospitalization [1,2,3]. Even though the mortality rate related to anaphylaxis has been low in the past few decades (less than 1%) [4], the management of anaphylaxis consists mainly of allergen avoidance, which can lead to increased stress, anxiety and even depression. Moreover, patients with a history of anaphylactic reactions have a significantly impaired quality of life [2]. Identifying the triggers and risk factors is crucial in the management of anaphylaxis, and aids in creating personalized management plans.

The diagnosis of anaphylaxis is traditionally based on clinical symptoms and patient history. However, these methods have limitations, particularly in distinguishing between immunoglobulin E (IgE)-mediated and non-IgE-mediated reactions or in cases with multiple allergens that may trigger severe reactions. Non-IgE-mediated allergic reactions are driven by components of the immune system other than IgE antibodies, such as T cells. Non-IgE-mediated reactions are usually delayed, and, in the case of non-IgE-mediated food allergy, mostly include symptoms such as vomiting, bloating and diarrhea [5]. In IgE-mediated reactions, IgE antibodies play a key role in the early type of allergic reactions and atopic allergic diseases. Allergen-specific IgE (sIgE) represents a crucial target (biomarker) for both diagnostic purposes and therapeutic approaches. In comparison with other immunoglobulin classes, IgE antibodies are of extremely low abundance, ranging from 1 to 400 ng/mL in non-atopic people, which is why it was the last of the immunoglobulin family to be discovered [6,7,8]. About 50% of IgE antibodies are free, and about 50% are bound to IgE receptors on the effector cells (blood basophils, tissue mast cells, antigen-presenting cells such as monocytes and macrophages, intestinal epithelial cells, airway epithelial and smooth muscle cells) [9]. Their effector functions are activated by IgE binding to high affinity receptor Fc epsilon receptor I (FcεRI) and by binding to the low affinity Fc epsilon receptor II/cluster of differentiation 23 (Fc receptor FcεRII/CD23) [10,11]. The cross-linking of FcεRI receptor-bound allergen-specific IgE on effector cells (mast cells and basophils) by allergen encounter is the signal for cell degranulation and release of mediators (e.g., histamine, chemotactic factors, leukotrienes, neurokinins, complement anaphylatoxins and others) causing severe allergic reactions, which manifest by anaphylactic shock and even death [8,9]. The cross-linking of only about 1% of IgE molecules on the surface of effector cells is required for half-maximal activation of the cell [12,13,14]. The half-life of free IgE is only a few days, while the half-life of IgE bound to cellular receptors is about 2 months. The activation of effector cells is influenced by numerous factors: the ratio of allergen-specific to total IgE, the proportion of bound IgE, the ratio of low- to high-affinity IgE antibodies, the total amount of epitope-specific antibodies (clonality), affinity of IgE antibodies and avidity of multivalent IgE binding sites. [15,16,17].

Many factors affect the severity of the allergic reaction, including those related to the allergen type or nature or the route of exposure, as well as individual sensitivity or susceptibility of the patient [15,18]. This heterogeneity contributes to clinicals dilemmas in the management of anaphylaxis, especially in patients at risk for recurrent systemic or severe allergic reactions, and patients in whom the findings of diagnostic tests are not consistent with the clinical picture [15,19].

In clinical practice, highly sensitive and specific in vitro tests are used to detect sIgE antibodies to a wide spectrum of allergens. The presence of sIgE antibodies to a particular allergen does not imply that results have any clinical relevance, especially in patients with anaphylaxis. The clinical significance and predictive value of these tests are critically dependent on the suggestive anamnesis related to a plausible trigger. In patients whose history of anaphylaxis to a specific trigger is inconclusive (e.g., patients with food allergy or insect sting allergy), the predictive value of sIgE tests is low, and these tests should not be used as screening tools [20].

This review provides insight into the clinical utility of the most commonly used in vitro assays in the diagnosis of anaphylaxis, including component-resolved diagnostics (CRD), both in singleplex and multiplex format.

## 2. Measurement of Specific IgE Antibodies

Most in vitro-specific IgE tests use standardized aqueous extracts obtained from natural sources, such as environmental inhaled allergens (tree pollen, grass, weeds, molds, epithelium or pet hair, house dust mites) or food. The choice of sIgE to be tested should always be determined by a careful clinical history, and both in vitro as well as in vivo tests (such as the skin prick test) should be applied as complementary tests in the diagnosis of anaphylaxis [21,22]. However, such allergen extracts comprise thousands of different macromolecules, and certain allergen molecules may be poorly represented. Additionally, the content of these extracts varies between manufacturers and even between batches. Traditional sIgE tests, including the gold standard solid phase immunoassay (ImmunoCAP™), although highly specific for the allergen type in question, detect sIgE antibodies to cross-reactive determinants, which may not be relevant, and therefore give rise to false positive results [21].

Advances in laboratory diagnostics in allergic diseases have led to the development of CRD tests that detect sIgE against molecularly defined allergenic components. CRD can distinguish between sIgE to primary allergenic molecules from cross-reactivity or sensitivity to allergens of similar molecular structure with greater certainty [22,23]. The use of individual allergenic molecules introduced the high-resolution molecular diagnostics into clinical practice in risk identification and stratification, treatment guidance, follow-up and prognostic evaluation of a range of allergic conditions, including anaphylaxis. However, due to the large allergomic data it provides, the utility of CRD in the diagnostics and management of anaphylaxis still requires careful interpretation and multidisciplinary clinical expertise [24,25,26].

## 3. CRD in the Management of Anaphylaxis

CRD plays an increasingly important role in the evaluation and management of anaphylaxis by enabling precise identification of sensitization and sensitization profiles to different allergenic components. This molecular approach supports improved risk stratification, particularly in distinguishing between clinically relevant sensitizations and cross-reactive, less meaningful ones. CRD is especially valuable in complex cases where the trigger is unclear, or multiple potential allergens are involved, setting the stage for more targeted investigation in food, insect venom or idiopathic anaphylaxis.

### 3.1. Food-Induced Anaphylaxis

CRD is widely used in the diagnosis and management of food-induced anaphylaxis. It is particularly useful in identifying specific allergenic proteins in foods like peanuts, tree nuts, milk, eggs, fish and other “major” allergens. In food allergy, sensitization to certain allergen components is associated with treatment outcomes, allergic disease prognosis and increased or decreased relative risk of severe reactions to the food from which it is derived. For example, in a study involving infants, the sensitivity of sIgE to Ara h 2 was 60% in correctly identifying children with true peanut allergy compared to only 26% correctly identified by using whole peanut sIgE [27]. Sensitization to Ara h 2 is a strong predictor of clinically relevant peanut allergy, with a positive predictive value (PPV) of around 90%, and is associated with a high risk of anaphylaxis to peanut, while Ara h 9 is associated with a low relative risk for the occurrence of anaphylaxis [28,29,30,31,32].

Similarly, nBos d 8 (cow’s milk casein) is a better marker of prediction to cow’s milk allergy than other components such as Bos d 4 (α-lactalbumin) or Bos d 5 (β-lactoglobulin) in pediatric patients. Additionally, an nBos d 8 level greater than 1.8 kUA/L increases the risk of anaphylaxis in children with cow’s milk allergy up to six times, with a specificity of 77% and sensitivity of 65% [33].

Gal d 1 (ovomucoid) is a major heat-stable egg allergen strongly associated with severe allergic reactions, including anaphylaxis. Studies have shown that elevated ovomucoid-specific IgE correlates with an increased risk of systemic reactions during oral food challenges. For example, a Gal d 1 cutoff of ≥10 kU/L had a specificity of 95% and PPV circa 90% for predicting more severe reactions, to both raw and cooked egg [34]. Sensitivity at this cutoff was, however, moderate (circa 50–60%), indicating that not all patients with severe allergy have high levels of sIgE to Gal d 1. Since Gal d 1 is heat-stable, it is able to predict the risk for anaphylaxis even after ingestion of baked or processed egg products, thus distinguishing persistent allergy from mild, transient egg sensitivity [35].

Cor a 9 (a 11S globulin seed storage protein) and Cor a 14 (a 2S albumin storage protein) are both linked to hazelnut allergy and anaphylactic reactions to hazelnut. Specifically, Cor a 9-specific IgE at a threshold of ≥1 kU/L exhibits approximately 80% sensitivity and over 90% specificity for predicting moderate to severe reactions to hazelnut, including anaphylaxis [36]. Furthermore, positive sensitization to Cor a 9 indicates a PPV of 60–70% for systemic reactions [36,37]. Conversely, negative results for Cor a 9, especially when combined with negative results for Cor a 14, yield a negative predictive value (NPV) over 85%, effectively ruling out severe hazelnut allergy in most patients. On the other hand, Cor a 1 exhibits a high degree of cross reactivity with its homologue Bet v 1 [38].

However, the relative risk for anaphylaxis associated with a specific allergen component does not always imply clinical relevance for individual patients. Clinical relevance is highly dependent on the patient’s history of previous anaphylactic reactions (especially severe ones) and the results of allergen challenge testing, with the gold standard being a double-blind placebo-controlled food challenge test (DBPCFC) [32,39]. Specific IgE testing to potential cross-reactive allergen components is mostly used in cases of symptoms that arise with multiple plant foods, in distinguishing true allergy versus oral allergic syndrome, latex allergy (due to certain fruits having similar proteins to latex) and in polysensitization to pollen allergens. In respiratory allergic diseases, CRD is useful in identifying true sensitization versus cross-reactive allergen components, and in guiding allergen-specific immunotherapy as well as monitoring its success. [32,39,40,41,42].

### 3.2. Insect Venom-Induced Anaphylaxis

In *Hymenoptera* venom allergy, CRD helps in identifying the specific venom components responsible for anaphylaxis. This is crucial for selecting the appropriate venom for immunotherapy and avoiding unnecessary treatment, which prolongs the course of immunotherapy and ultimately leads to its decreased efficiency due to poorer adherence [43]. CRD also helps in distinguishing between primary sensitization and cross-reactivity, which is common in patients sensitized to both honeybee and wasp venom [44]. Using Ves v 5 in isolated wasp allergy has a sensitivity of 78.5%, and when it is combined with Ves v 1, the sensitivity increases to 92.3%. However, the sensitivity of Api m 1 was only 25% for isolated honey bee allergy. [45]. Compared to whole allergen extracts, CRD can better discriminate true sensitization to honeybee venom versus wasp/yellow jacket venom (Api m 1, Api m 3, Api m 4 and Api m 10 vs. Ves v 1/Pol d 1 and Ves v 5/Pol d 5) from cross-reactivity between insect species (hyaluronidases Api m 2, Ves v 2 and Pol d 2 as well as dipeptidyl peptidases IV- Api m 5, Ves v 3 and Pol d 3) [46,47].

### 3.3. Latex-Induced Anaphylaxis

CRD is also a reliable tool for diagnosing latex allergy. Using CRD, especially in multiplex arrays, can enhance the diagnosis of IgE-mediated allergy to latex by discriminating between true allergy and sensitization. For example, the presence of Hev b 5 and Hev b 6.02 sIgE can be a strong indicator of clinically significant latex allergy and help guide management decisions. A study involving participants with occupational asthma found that for Hev b 5 and Hev b 6.02 combined, the sensitivity was 79%, with a specificity of 88%, PPV was 96% and NPV was 56% [48]. By focusing on specific components, CRD reduces the likelihood of false positives, which can occur when sIgE tests detect antibodies against cross-reactive carbohydrate determinants (CCDs) or profilin, which can lead to a positive test result without actual latex allergy. Interestingly, Hev b 6.02 (hevein) shows a high level of cross-reactivity to avocado, which may also aid in resolving potential latex allergy [49].

### 3.4. Idiopathic Anaphylaxis

In cases of idiopathic anaphylaxis, where the causative allergen is unknown, CRD can help identify potential allergens that may have been missed by conventional diagnostic methods. For example, CRD can detect sensitization to galactose-α-1,3-galactose (α-gal), a red meat allergen, which is also found in the saliva of certain ticks and is responsible for the alpha-gal syndrome [50]. In a study involving different populations, 55% of patients with idiopathic anaphylaxis were later identified as having allergy to ω-5-gliadin, indicating a significant underestimation of this allergy [51]. Studies have shown that up to 20% of idiopathic anaphylaxis may be resolved using CRD, especially with multiplex arrays [52,53,54].

### 3.5. Cross-Reactive Carbohydrate Determinants

CCDs are N glycan carbohydrate moieties, such as the α 1,3 linked fucose on core N acetylglucosamine, and are common in allergen sources. They often cause cross-reactivity in IgE testing, for example in honeybee and wasp venom cross-reactivity, but have poor correlation with clinical symptoms. Using sIgE with CCDs, especially in combination with other allergen components, is useful in identifying true sensitization to specific insect venom and helping avoid unnecessary immunotherapy treatment [47]. Certain tests use CCD inhibitors during serum processing, which substantially reduces false positives and is particularly useful when interpreting unclear allergen profiles [55].

CRD is also essential in allergy diagnostics, especially in cases of pollen-associated food allergy and oral allergy syndrome. With their utilization clinicians get a better insight into a patient’s sensitization profile and help tailor disease management and preventive measures, such as food avoidance. The patient’s sensitization profile and known allergen (allergen component) concentration in CRD may help to estimate symptom severity and risk of anaphylaxis.

## 4. Measurement of Specific IgE Antibodies to Allergen Molecules in Singleplex Assays

Since the introduction of CRD and the establishment of the allergome, many specific molecular allergen components from numerous allergen sources, including food, inhalant sources, insect venom and others, have been identified and reported in clinical practice. This has allowed for the identification of dominant or major allergen components, which are commonly recognized by IgE antibodies in the sera of allergic patients. Certain allergen components are purified from natural sources and usually contain multiple naturally-occurring isoforms of the allergen, and a range of epitopes as well as CCDs. Other allergen components are present in very small quantities in the natural allergen source but might still have potent allergenicity and be clinically relevant. Such components are usually recombinantly produced to increase their quantities and uniformity to accurately design a sIgE test [24,30]. In fact, the introduction of singleplex CRD tests has proven useful in the management of anaphylaxis due to the increase in analytical sensitivity of the tests, especially when crucial allergens are unstable or poorly represented in allergen extracts and in the increase in analytical specificity. This is especially important in risk assessment, e.g., in patients with food allergy in whom sensitization to specific allergen components is associated with higher risk of severe anaphylactic reactions and in identifying cross-reactivity, both of which significantly impact the management of allergic diseases and guide prevention strategies [30,31]. However, successful utilization of these tests requires careful choice of the sIgE based on individual patient history, skin prick testing, results of the tests using crude allergen extracts, clinical relevance and availability of the allergen components [31].

## 5. Measurement of Specific IgE Antibodies in Multiplex Assays

In the past decade, multiplex assays have been used for the determination of specific IgE-sensitization to a large number of allergen components. Multiplex tests allow for the simultaneous detection of IgE antibodies to a range of allergens, reducing the need for a large number of separate tests. This is particularly advantageous in cases of anaphylaxis where multiple allergens, such as foods, insect stings and medications, may be involved. By combining numerous allergen-specific IgE tests into a single assay, multiplex testing may be more cost-effective and certainly more time-efficient than conducting individual tests. This is particularly beneficial in anaphylaxis, where rapid diagnosis and management are essential. Multiple testing provides a comprehensive allergen profile that can help identify multiple potential triggers of anaphylaxis. This is especially important in cases where the history of exposure to specific allergens is unclear or when the patient has a history of reactions to a wide variety of substances [22,23,24]. Additionally, multiplex sIgE assays are especially useful in cases of polysensitization to food allergens, where patients have to follow an extremely restrictive diet, in distinguishing patients with oral allergy syndrome versus true food allergy and in patients with multimorbid allergic phenotypes [21,23,24].

There are several multiplex sIgE assays available at this moment, including those with a broad spectrum of allergen sources, such as the ISAC 112 (Immuno Solid-phase Allergen Chip, Thermo Fisher Scientific, Uppsala, Sweden, available since 2008) and ALEX (Allergy Explorer, Macro Array Diagnostics, Vienna, Austria) platforms, as well as those with targeted allergen source groups (e.g., the EUROLINE DPA-DX immunoassays). ISAC includes 112 inhaled and food allergen components and is currently the most frequently used and studied multiplex assay in clinical practice. In 2019 a novel multiplex array in molecular allergy diagnostics, ALEX, was launched and became commercially available. The ALEX multiplex array represents an extended platform encompassing 117 allergen extracts and 178 molecular components (ALEX2) from inhaled, food, animal, latex and insect allergen sources. The presentation of common clinical indications where multiplex testing is more or less recommended are summarized in Table 1 [54,56,57,58,59].

The ISAC and ALEX assays are comparable with each other to a certain extent. A pilot study that examined the agreement between the ALEX and ISAC platforms included a limited number of atopic children with multiple food allergies. It showed a good agreement for all major allergens: egg, cow’s milk, peanut, codfish/prawn, soy and wheat components. The negative percent agreement between ISAC and ALEX was better than the positive percent agreement (90.3 and 79.5, respectively) [60]. In another study involving 49 patients, the levels of positive and negative percentage agreement between the ISAC and ALEX arrays were 90% and 95%, respectively. High negative and positive percentage agreement was found for egg, milk, storage proteins (nuts, seeds and legumes), grass, tree, animals, mites, molds and cross-reactive allergens (lipid transfer proteins, LTPs, pathogenesis-related-10 (PR-10), profilins, tropomyosins and albumins). Good agreement was found among animal epithelia, grass pollen and the PR-10 protein. The study revealed low agreement for the Niemann-Pick type C2 (NPC2) family of Blomia and Lepidoglyphus, but a good correlation for the same family of mites (*Dermatophagoides farina* and *D. pteronyssinus*). Among tree pollen components, olive pollen (Ole e 9) was positive in ISAC but found to be negative in the ALEX assay. Certain discrepancies were found in storage protein components, which are usually heat- and digestion-stable and responsible for the most severe reactions. Additionally, the ISAC platform seemed to be less sensitive at very low reactivity for the major allergen Ara h 2 compared to the ALEX test [61]. A recent brief report conducted in central Spain involving patients (both children and adults) with suspected lipid transfer protein (LTP)-syndrome who were polysensitized showed poor agreement in ALEX2 compared to ISAC for Ole e 7. With regards to nut LTPs, the performance of Jug r 3 was poor in patients with low levels of sensitization or high sensitization levels with low total serum IgE amounts. Patients who tested positive in the ISAC but negative in the ALEX2 assay concerning Jug r 3 had a total IgE amount below 100 kU/L. However, in patients with high levels of sensitization, the performance of Jug r 3 was better. Moreover, the ALEX2 test, unlike ISAC, incorporates the tomato component Sola l 6. [62]. A study involving patients with allergic diseases (rhinitis, bronchial asthma and/or atopic dermatitis) aged 1–79 years examined species-specific components, plant and animal panallergens and CCDs, comparing all allergens and molecules shared by the two platforms (a total of 102 components). A high correlation was found for the main indoor and outdoor allergens: Alt a 1, Cup a 1, Der f 1 and 2, Der p 2 and 23, and Par p 1 and Phl p 5. A very high correlation was found for Der p 1. Non-specific lipid transfer proteins (nsLTPs), profilins, PR-10 and polcalcins were studied as panallergens. A significant correlation was found for almost all nsLTP components except Tri a 14 and Ole e 7. Jug r 3, Mal d 3, and Pru p 3 were more frequently detected by the ISAC platform. Profilin evaluation revealed strong concordance between platforms, especially in the case of Phl p 12 [63]. The main difference between these two assays is that ALEX2 encompasses insect venom components and extracts, giving it a comparative advantage in the diagnosis of anaphylaxis. Table 2 shows the comparison of components included in these two platforms.

Although multiplex tests provide a several-fold larger amount of clinical information compared to singleplex assays, they require even more careful clinical interpretation and expertise. Additionally, they are not commonly used as screening tests for allergy because even though they include over 100 allergen components and extracts, the number of these components is still limited in comparison to the entire allergome. Additionally, multiplex assays are often less sensitive compared to singleplex tests; moreover, a large proportion of positive findings in such tests are likely clinically irrelevant in most cases. Finally, multiplex assays are still rather expensive and not easily available in all countries of the world [64].

## 6. Future Directions in CRD

Recently, efforts have been made to further enhance CRD to improve patient outcomes and reduce the burden of anaphylaxis. These novel approaches include the integration of CRD findings with other diagnostic tests and procedures into complex prediction models using machine learning and artificial intelligence tools. With algorithms based on machine learning and artificial intelligence, healthcare providers are able to analyze very large patients datasets, which may enable them to identify patterns that are not immediately visible even to highly experienced clinicians. These algorithms can thus help to identify diseases and risks for anaphylaxis earlier, resulting in expedited medical intervention and improved clinical outcomes [65]. Novel studies show that utilizing the basophil activation test (BAT) in combination with CRD increases the risk predictive power in food allergy [66,67]. Similarly, using the emerging mast cell activation test (MAT) to specific allergen components may enhance the accuracy in identifying key triggers of anaphylaxis [68]. Novel findings indicate that using genetic screening tests for common variations in the receptor tyrosine kinase (KIT p.D816V) in combination with other assays, including CRD, identifies clonal mast cell expansion in patients with insect venom allergy at high risk of severe anaphylactic reactions [69]. With the growing use of artificial intelligence tools, even in clinical settings, utilizing AI tools as an aid in the interpretation of complex CRD findings may enable a widespread implementation of CRD assays globally, facilitating their standardization and intercomparison between different platforms [70]. These new approaches will revolutionize the use of CRD in clinical practice and enable completely individually tailored treatment strategies, improve anaphylaxis outcomes and reduce the risk of adverse reactions during immunotherapy [71].

## 7. Conclusions

Anaphylaxis is a severe, life-threatening allergic reaction that requires prompt diagnosis and identification of key culprits. CRD is a precise tool for in vitro allergy diagnostics, which significantly improved the quality of anaphylaxis management. By offering greater specificity, improved sensitivity, and the ability to identify multiple allergens simultaneously, CRD enabled clinicians to distinguish true allergy/sensitization from cross-reactions, identify major allergen components associated with higher risks of severe anaphylactic reactions, help select patients eligible for allergen immunotherapy, as well guide immunotherapy regimes. This paved the way for the implementation of a personalized approach in the diagnosis of anaphylaxis. A large number of individual sIgE tests for allergen components and several multiplex platforms are now available, providing detailed information and large amounts of clinical data, improving decision making in the context of precision medicine and even resolving cases that were previously categorized as idiopathic anaphylaxis. However, their widespread implementation will require overcoming challenges such as cost, availability and the need for expert interpretation, as these multiplex assays are only comparable to each other to a certain extent, and more importantly, they generate huge datasets, with many of the positive findings in such assays likely being clinically irrelevant. New tools and approaches, such as the utilization of complex prediction models involving other diagnostic tests and procedures, as well as artificial intelligence, could simplify the process of the interpretation of CRD results and ensure individually tailored, accurate, and timely management of anaphylaxis, ultimately improving patient outcomes and their quality of life.

## Figures and Tables

**Table 1:** The most common clinical manifestations of allergic diseases when multiplex sIgE tests are recommended or not recommended. * for ISAC test. sIgE—allergen specific immunoglobulin E, IgE—immunoglobulin E, ISAC—Immuno Solid-phase Allergen Chip.

Multiplex sIgE Testing Recommended	Consider Other Diagnostic Tests Prior to Multiplex sIgE Testing	Multiple sIgE Testing Not Recommended
Investigation in patients with anaphylaxis to unknown triggers	Anaphylaxis, suspected venom allergen trigger that can be measured by sIgE, or singleplex assay	Suspected drug allergy—no drugs are tested in multiplex tests
Objective assessment of clinical relevance of allergy in children or adults on highly restrictive elimination diets	Suspected IgE-mediated food allergy; consider singleplex assays if one or several potential allergens are in question; consider multiplex assays if a large number of sIgE needs to be tested (e.g., 10 or more)	Assessment of potential insect venom allergy (not included in the multiplex test) *
Diagnostics in patients with oral allergy syndrome	Suspected mammalian meat allergy. Consider measurement of specific IgE for α-gal and milk, beef, pork and lamb.	Assessment of progress or diagnosis of allergy to single food allergens, e.g., milk, peanut, egg. Specific IgE measurement in singleplex format is preferred.
Detailed assessment in known polysensitized and patients with multi-morbid allergic phenotypes, for detection of dominant sensitization/triggers in guiding allergen immunotherapy	Moderate to severe atopic dermatitis with suspected food allergy. Assess whether allergic reactions are IgE-mediated.	Atopic dermatitis—for investigation of allergic contributions (if total serum IgE is low). Consider measurement of specific IgE for common inhalant allergens and major or selected food allergens

**Table 2:** A comparison of allergen components included in the ISAC and ALEX2 platforms. * research only. YES—the same allergen component is included in the ALEX2 platform and ISAC platform. nsLTP—non-specific lipid transfer protein, NPC2—Niemann-Pick type C2, PR-10—pathogenesis-related-10, CCD—cross-reactive carbohydrate determinant.

Allergen Source	ISAC	ALEX2	Protein Type
**Mainly species-specific food**	**Allergen component**		
egg white	Gal d 1	YES	ovomucoid
	Gal d 2	YES	ovalbumin
	Gal d 3	YES	ovalbumin/ovotransferrin
	Not included	Gal d 4	lysozyme C
egg yolk/chicken	Gal d 5	YES	livetin/serum albumin
cow’s milk	Bos d 4	YES	alpha-lactalbumin
	Bos d 5	YES	beta-lactoglobulin,
	Bos d 8	YES	casein
	Bos d lactoferrin	Not included	transferrin
cow/meat	Not included	Bos d 2	lipocalin
Alpha-Gal	Alpha-Gal	Not included	galactose-alpha-1,3-galactose
buckwheat	Fag e 2	YES	storage protein, 2S albumin
Baltic cod	Gad c 1	Not included	parvalbumin (beta)
Atlantic cod	Not included	Gad m 1	parvalbumin (beta)
Atlantic cod	Not included	Gad m 2+3	enolase (beta)/aldolase
shrimp	Not included	Pen m 1	tropomyosin
	Pen m 2	YES	arginine kinase
	Not included	Pen m 3	myosin light chain
	Pen m 4	YES	sarcoplasmic calcium-binding protein
Atlantic herring	Not included	Clu h 1	parvalbumin (beta)
brown shrimp	Not included	Cra c 6	troponin C
carp	Not included	Cyp c 1	parvalbumin (beta)
thornback ray	Not included	Raj c parvalbumin	parvalbumin (alpha)
salmon	Not included	Sal s 1	parvalbumin (beta)
Atlantic mackerel	Not included	Sco s 1	parvalbumin (beta)
tuna	Not included	Thu a 1	parvalbumin (beta)
swordfish	Not included	Xip g 1	parvalbumin (beta)
cashew nut	Ana o 2	YES	storage protein, 11S globulin
	Ana o 3	YES	storage protein, 2S albumin
Brazil nut	Ber e 1	YES	storage protein, 2S albumin
hazelnut	Cor a 9	YES	storage protein, 11S globulin
	Cor a 14	YES	storage protein, 2S albumin
	Not included	Cor a 11	storage protein, 7/8 S globulin
walnut	Jug r 1	YES	storage protein, 2S albumin
	Not included	Jug r 2	storage protein, 7/8 S globulin
	Not included	Jug r 3	storage protein, 2S albumin
	Not included	Jug r 4	storage protein, 11S albumin
	Not included	Jug r 6	storage protein, 7/8 S globulin
sesame seed	Ses I 1	YES	storage protein, 2S albumin
peanut	Ara h 1	YES	storage protein, 7S globulin
	Ara h 2	YES	storage protein, 2S albumin
	Ara h 3	YES	storage protein, 11S globulin
	Ara h 6	YES	storage protein, 2S albumin
	Not included	Ara h 15	oleosin
soybean	Gly m 5	YES	storage protein, beta-conglycinin
	Gly m 6	YES	storage protein, glycinin
	Not included	Gly m 8	storage protein, 2S albumin
	Tri a 19.0101	YES	omega-5 gliadin
	Tri a 40.0101	YES	alpha-amylase/trypsin inhibitor
kiwi	Act d 1	YES	cysteine protease
	Not included	Act d 2	thaumatin-like protein
apple	Not included	Mal d 2	thaumatin-like protein
kiwi	Act d 5	YES	kiwellin
macadamia nut	Not included	Mac i 2	storage protein, 2S albumin
mustard	Not included	Sin a 1	storage protein, 2S albumin
pistachio	Not included	Pis v 1	storage protein, 2S albumin
	Not included	Pis v 2	11S globulin subunit
	Not included	Pis v 3	storage protein, 7/8 S globulin
poppy seed	Not included	Pap s 2	storage protein, 2S albumin
**Mainly species-specific aeroallergen**			
Bermuda grass	Cyn d 1	YES + extract	grass group 1
timothy grass	Phl p 1	YES	grass group 1
	Phl p 2	YES	grass group 2
	Phl p 4	Not included	berberine bridge enzyme
	Phl p 5	YES	grass group 5
	Phl p 6	YES	grass group 6
	Phl p 11	Not included	ole e 1-related protein
	Not included	Phl p 12	profilin
perennial ryegrass	Not included	Lol p 1	beta-expansin
birch	Bet v 1	YES	PR-10 protein
birch	Not included	Bet v 6	isoflavone reductase
Japanese cedar	Cry j 1	YES	pectate lyase
cypress	Cup a 1	YES	pectate lyase
ash	Not included	Fra e 1	Common Olive Group 1
olive pollen	Ole e 1	YES	Common Olive Group 1
	Ole e 9	YES	beta-1,3-glucanase
plane tree	Pla a 1	YES	putative invertase inhibitor
plane tree	Not included	Pla a 2	polygalacturonase
ragweed	Amb a 1	YES	pectate lyase
ragweed	Not included	Amb a 4	defensin
mugwort	Art v 1	YES	defensin
goosefoot	Che a 1	YES	ole e 1-related protein
wall pellitory	Par j 2	YES	nsLTP
plantain	Pla l 1	YES	ole e 1-related protein
saltwort	Sal k 1	YES	pectin methylesterase
dog	Can f 1	YES	lipocalin
	Can f 2	YES	lipocalin
	Can f 4	YES	lipocalin
	Can f 5	YES	arginine esterase
	Can f 6	YES	lipocalin
	Not included	Can f_Fd 1	uteroglobin
horse	Equ c 1	YES	lipocalin
	Not included	Equ c 4	laterin
cat	Fel d 1	YES	uteroglobin
	Not included	Fel d 2	lipocalin
	Fel d 4	YES	lipocalin
cat	Not included	Fel d 7	lipocalin
mouse	Mus m 1	YES	lipocalin
rabbit	Not included	Ory c 1	lipocalin
	Not included	Ory c 2	lipocalin
rabbit	Not included	Ory c 3	uteroglobin
Djungarian hamster	Not included	Phod s 1	lipocalin
cockroach	Bla g 1	YES	cockroach group 1
	Bla g 2	YES	aspartic protease
	Not included	Bla g 4	lipocalin
	Bla g 5	YES	glutathione S-transferase
	Not included	Bla g 9	arginine kinase
American cockroach	Not included	Per a 7	tropomyosin
Alternaria sp.	Alt a 1	YES	acidic glycoprotein
	Alt a 6	YES	enolase
Aspergillus sp.	Asp f 1	YES	mitogillin family
	Asp f 3	YES	peroxisomal protein
	Not included	Asp f 4	unknown
	Asp f 6	YES	Mn superoxide dismutase
Cladosporium sp.	Cla h 8	YES	mannitol dehydrogenase
M. sympodial	Not included	Mala s 5	unknown
	Not included	Mala s 6	cyclophilin
	Not included	Mala s 11	Mn superoxide dismutase
B. tropicalis	Blo t 5	YES	mite group 5
B. tropicalis	Not included	Blo t 10	tropomyosin
B. tropicalis	Not included	Blo t 21	unknown
D. farinae	Der f 1	YES	cysteine protease
	Der f 2	YES	NPC2 family
D. pteronyssinus	Der p 1	YES	cysteine protease
	Der p 2	YES	NPC2 family
D. pteronyssinus	Not included	Der p 5	unknown
	Not included	Der f 7	mite group 7
	Not included	Der f 10	tropomyosin
	Not included	Der p 11	myosin, heavy chain
	Not included	Der p 20	arginine kinase
	Der p 23	YES	peritrophin-like protein domain
G. domestica	Not included	Gly d 2	NPC2 family
L. destructor	Lep d 2	YES	NPC2 family
L. destructor	Not included	Tyr p 2	NPC2 family
**Other mainly species-specific components**			
Anisakis	Ani s 1	YES	serine protease inhibitor
	Not included	Ani s 3	tropomyosin
pigeon tick	Not included	Arg r 1	lipocalin
latex	Hev b 1	YES	rubber elongation factor
	Hev b 3	YES	small rubber particle protein
	Hev b 5	YES	acidic protein
	Hev b 6	YES	hevein
	Not included	Hev b 11	class 1 chitinase
**Insect venom components**			
Honey bee venom	Not included	Api m 1	phospholipase A2
	Not included	Api m 10	icarapin Variant 2
Paper wasp venom	Not included	Pol d 5	antigen 5
Common wasp venom	Not included	Ves v 1	phospholipase A1
	Not included	Ves v 5	antigen 5
**Cross-reactive components**			
cow’s milk	Bos d 6	YES	serum albumin
dog	Can f 3	YES	serum albumin
horse	Equ c 3	YES	serum albumin
cat	Fel d 2	YES	serum albumin
pig	Not included	Sus d 1	serum albumin
Anisakis	Ani s 3	YES	tropomyosin
cockroach	Bla g 7	YES	tropomyosin
D. pteronyssinus	Der p 10	YES	tropomyosin
shrimp	Pen m 1	YES	tropomyosin
peanut	Ara h 9	YES	nsLTP
hazelnut	Cor a 8	YES	nsLTP
hemp	Not included	Can s 3	nsLTP
peach	Pru p 3	YES	nsLTP
mugwort	Art v 3	YES	nsLTP
olive pollen	Ole e 7	YES *	nsLTP
plane tree	Pla a 3	YES	nsLTP
corn	Not included	Zea m 14	nsLTP
wheat	Tri a 14	YES	nsLTP
kiwi	Not included	Act d 10	nsLTP
apple	Not included	Mal d 3	nsLTP
Vitis vinifera	Not included	Vit v 1	nsLTP
celery	Not included	Api g 2	nsLTP
celery	Not included	Api g 6	nsLTP
tomato	Not included	Sola l 6	nsLTP
birch	Bet v 1	YES	PR-10 protein
alder	Aln g 1	YES	PR-10 protein
hazel pollen	Cor a 1.0101	NO	PR-10 protein
hazelnut	Cor a 1.0401	YES	PR-10 protein
hazel pollen	Not included	Cor a 1.0103	PR-10 protein
beech	Not included	Fag f 1	PR-10 protein
apple	Mal d 1	YES	PR-10 protein
peach	Pru p 1	Not included	PR-10 protein
soybean	Gly m 4	YES	PR-10 protein
peanut	Ara h 8	YES	PR-10 protein
kiwi	Act d 8	Not included	PR-10 protein
celery	Api g 1	YES	PR-10 protein
strawberry	Not included	Fra a 1+3	PR-10 protein + LTP
carrot	Not included	Dau c 1	PR-10 protein
kiwi	Act d 2	YES	thaumatine-like protein
birch	Bet v 2	YES	profilin
latex	Hev b 8	YES	profilin
annual mercury	Mer a 1	YES	profilin
timothy grass	Phl p 12	YES	profilin
date palm	Not included	Pho d 2	profilin
muskmelon	Not included	Cuc m 2	profilin
birch	Bet v 4	Not included	polcalcin
timothy grass	Phl p 7	YES	polcalcin
alder	Not included	Aln g 4	polcalcin
bromelain	Not included	MUXF3	CCD
NO	lactoferrin	Hom s LF	CCD

## Data Availability

No new data were created or analyzed in this study. Data sharing is not applicable to this article.

## Abbreviations

The following abbreviations are used in this manuscript:

BAT	Basophil activation test
CCD	Cross-reactive carbohydrate determinant
CD23	Cluster of differentiation 23
CRD	Component-resolved diagnostics
DBPCFC	Double-blind placebo-controlled food challenge test
FcεRI	Fc epsilon receptor I
FcεRII	Fc epsilon receptor II
IgE	Immunoglobulin E
LTP	Lipid transfer protein
MAT	Mast cell activation test
NPV	Negative predictive value
nsLTP	Non-specific lipid transfer protein
PPV	Positive predictive value
PR-10	Pathogenesis-related 10
sIgE	Allergen-specific immunoglobulin E
